# Dr. Coxe, on Vaccination

**Published:** 1805-01-01

**Authors:** John Redman Coxe


*Copy of a Letter from Dr. Coxe, of Philadelphia, to Mr. Ring, dated October* 26, 1804.

My dear Sir,

YOU have my sincere thanks for the additional favors you have conferred on me, by the transmission of the late Treatises on the subject of Vaccination. I received them a day or two past, and have just given them a cursory reading. It appears to me, that you have amply vindicated the honour of the practice; nor can I well imagine, on what principle Mr. Goldson would attempt to overturn the public confidence in it, by a detail of a few, at best, imperfect histories, of both the (supposed) diseases under his care. It has forcibly struck me, from the hasty perusal of his pamphlet, that he can have paid but small attention to the progress of this beautiful affection, and to its various anomalies. He has said much upon the difference in colour between the *natural* and *inoculated* disease, as if this could possibly influence its prophylactic property! What is it in the cow, in fact, but an inoculated disease from the heels of the horse? According to his principles, to insure *perpetual* safety, and not for *two* or

*three*

*three* years only, we had better recur at once to the equine as the primary, and consequently the most efficacious, source. But, if so great a falling off exists between the efficacy of the disease immediately from the cow and that produced by inoculation from the human subject, alas! how dangerously must a large proportion of the rising generation be now situated, from the *increased deterioration* of the infection by some hundred removes from its original stock! After all, Mr. Goldson has built his faith too much upon the early opinions respecting the disease, before it was thoroughly understood; and of course, (as might have been expected, and as he would have discovered by reading) many of them have been proved to be erroneous. With respect to this colour of the pock above adverted to, it is an anomaly no more to be accounted for, than many others which take place in this disease. It is certain, that at times the vesicle will be of a pellucid appearance; whilst, at others, it is nearly of an opaque white, resembling paper: yet, in both instances, preserving the characteristic depression and circular appearance; the virus also being equally transparent in both. In all the cases which have come under my care, and to which, from the interest I take in the practice, I have paid great attention, I have never met with but one instance of the real blue colour. I have, in two or three others, thought I perceived a distant approach to it. As this case serves to evince the variety of the disease, I shall just state the circumstances.

On the 27th of last November I vaccinated a young man, a student of medicine, by two punctures of the left arm (having previously attempted to infect him with some virus of five months old). It succeeded in *one* puncture only; and throughout its course appeared to be about two days *tardy*: thus, the areola did not occur until the eleventh day. The fever was rather high on the twelfth day, when he told me, he found, by examination, his pulse beat from ten to fifteen strokes more in a minute in the *sound* arm than in the other. I could not, however, find any difference on examining it. On the 2d of January I tested him with small-pox; and he has himself since, repeatedly exposed and inoculated himself with only a very slight inflammation of the part occurring. The infection I employed was some on glass, which Mr. G. C. Jenner gave to young Mr. Peale, when he returned from exhibiting the mammoth in England. It was two months and a half old when I employed it. There are some points in the the preceding case, which, compared with the next I shall state, show the diversity of the disease. I have omitted to say, that by puncture the vesicle was one of the most *oval* I have seen, and of the colour I have mentioned above; of an *opaque white*. On the *tenth* day I revaccinated him from himself, which took effect, and progressed as it ought, the areola commencing a few hours after that of the primary pock.

On the 6th of December, I vaccinated an infant of four months old, with fresh infection (from the case above-mentioned) of the eleventh day; though, from its *tardy* progress, it was not above the ninth in the natural course of the disease. I must state, that this child had been previously vaccinated, ineffectually, three times; with recent infection, with some of eight days, and with some of seven months. Both punctures, in this instance, took effect; and though produced by infection from a pock so tardy, it ran an opposite course; a slight fever was perceptible on the sixth day, and the areola made its appearance late on the 7th, or very early on the 8th. On the tenth the areola was rapidly declining. The scabs came off on the 19th and twentieth days. It was on the eighth day, the mother herself called my attention to the pocks, by asking me, what was the matter with them, they had altered so much in colour? They had, from the earliest appearance, a slight tinge of blue, but they had not particularly attracted my attention till the sixth or seventh day; from which time they increased most rapidly, so that now they were of a considerably deep indigo appearance. Had I not known the circumstance, relative to the occasional livid appearance, I should have been very uneasy; but I beheld in them only a beautiful variety of the disease, which I had long anxiously looked for. The mother herself was frightened, as some of the neighbours had told her, they looked as if they would mortify. This child I revaccinated on the sixth day, from herself, ineffectually.

Now, in these two instances, the extreme variety of the disease is well evinced. Infection from a tardy pock produces one of rapid progress; and from one of an opaque white hue is induced the highest grade of blue. How will Mr. Goldson explain these varieties? I cannot. In proof of the mildness of the disease, I may state, that the subject of the last case, during its progress, not only cut several teeth, but had also a most violent cold, which threatened threatened an attack of cynanche trachealis; yet without any additional increase in the symptoms of the vaccine.

A circumstance in the first case stated, leads me to observe how much attention is essential in this disease; I mean, the *success* of one puncture and failure of the other. Now, had I unfortunately made *but the unsuccessful attempt,* I should probably have thrown away the infection as effete; although I have long known, that it may fail in one instance, whilst it succeeds in another; nay, that the same infection which fails to day, will succeed in the identical patients five or six days later;* and if five or six days, why not at a longer period?

In the first trial of the disease which succeeded in this city, viz. on myself, of three portions of thread deposited in my left fore-arm, about an inch from each other, in a straight line to the hand, the *two* extreme ones took effect, whilst the *middle* one failed;† and repeatedly has the same infection failed in one case, which has succeeded in another. How is this? we are yet in the dark as to many of the laws of this virus upon the animal body. We must resolve it, in the latter case, to a constitutional indisposition; may we be allowed, in the former, to suppose a *local* indisposition to exist? My own case, as well as the first case detailed in this letter, seems to authorize it.

I have within these few days, repeated the *sixteenth* unsuccessful attempt to induce the vaccine in a child. I began with it in April last, when two months old; and have continued at intervals to repeat it, with infection *four* times recent, five times of one day old, once of two days, once of three, once of seven, once of eight, once of seventeen, once of twenty-two, and once of sixty-two days. A spurious pustule was produced on the first and seventh attempts; the infection being recent, and one day old, and taken on the seventh and eighth of the disease.‡ I am hence led to state, that although we may be confident of the genuine nature of our infection, it by no means follows that it will invariably produce the perfect disease. Too much stress has I think been laid upon this; for although spurious infection cannot produce a perfect disease; yet, certainly, by some peculiarity of constitution, or

* See note, p. 41, of my Treatise on Vaccination.

† See Case I. ibid.

or some early injury, a spurious affection will not unfrequently follow the deposition of the most perfect infection. I have had more than one instance, in which the same infection, deposited the same day, in different persons' arms, has failed in one, produced a perfect pock in a second, and a spurious one in a third.

The chief object, then, of the practitioner, must assuredly be, to make himself acquainted with the real and genuine appearance of the disease, as a standard; a deviation from which should always excite a proportionate suspicion. Had Mr. Goldson adverted to this truth, it is probable he would not have laid so much stress upon the source of the infection first employed. As Mr. G. appears to have never seen the vaccine disease before he practiced it himself, it is very extraordinary that a philosophic curiosity should not have led him to test some of his first cases *immediately*; especially, as he appears to have so great a share of ill-timed scepticism. Six cases, even if better substantiated than his, would bear but a small proportion to, I might now say, the millions who have been shielded by the Jennerian Egis. Were it not a preservative, instead of one solitary *suspected* case, how many thousands might not the adversaries of this invaluable blessing adduce, without fear of contradiction! But "they lie wait privily;" and, by this underhand, insidious conduct, endeavour to destroy the confidence of the public in the practice. The rash assertion of an illiterate or malicious individual, is to overthrow the matured and long experienced judgement of the brightest ornaments of the profession in every part of the world!

In December, 1801, about seven weeks after the introduction of the practice into Philadelphia, I vaccinated my infant son of three weeks old; since christened by the name of Edward Jenner Coxe. He passed through the disease in the most favourable manner. About three weeks from the insertion of the vaccine infection, I inoculated him for the small-pox; and repeated it in a week more. I again attempted it on the 18th of February, with the like unsuccessful issue. In February of the following year (or *fourteen months*; a period after vaccination, at which Mr. Goldson found strong constitutional symptoms) I renewed my attempts; and again on April 29, for the fifth time. January 31 and May 29 of the present year, I again tried the small-pox; and his arm is *now* slightly inflamed by an eighth attempt made on the 20th of the month. In all I have made about fourteen punctures; and always employed employed *recent* virus, taken from persons labouring under the natural small-pox, generally very severe. To this, I may add, that he has been exposed to the natural contagion by visiting persons in it; and by being held in their arms for a quarter of an hour at a time, at least a dozen times. Many have I inoculated once, ineffectually; and several two and three times. I have an infant daughter, now about ten weeks old, who was vaccinated at two weeks, and passed through the disease with scarcely a moment's indisposition. The scab was off the sixteenth day; and she, in like manner, has resisted the variolous inoculation of the 20th instant. In the spring of 1803, I tested about twenty persons of different ages and sexes, who had gone through the vaccine, from its first introduction up to the period alluded to. The infection I used was taken from a man who was covered with it, in the natural way. I employed it on them all, in the space of a few days. Of this number, not one escaped evincing the *activity* of the poison. In about one-third, a small inflammation, without any vesicle, continued nearly three or four days, with much itching, and then died away. In an equal number, perhaps, the local affection produced a small vesicle of lymph, which dried away in about six or seven days. But in the third division, a complete pustule formed; which regularly maturated, and from which, as in Mr. Goldson's fourth case, I could have produced the small-pox in as many, I doubt not, as I should have tried it in; provided they had not previously been secured against it. In all these last cases, (and some had two or three pustules, according to the number of punctures made) no constitutional symptoms occurred; and though the pustule was surrounded with an inflamed and hardened base, no confluent pimples attended, but the disease was almost circumscribed as in the vaccine itself. In about one hundred persons whom I have tested, I have not been able to produce the small-pox, further than this slight local affection; and I will ask Mr. Goldson, if he would not consider it very unfortunate indeed, to fail so uniformly, without having the same invaluable cause to assign for it? I must therefore think, that Mr. Goldson might be much better employed, in studying the nature of vaccination, than in starting objections to the practice, founded on a few such imperfect experiments as he details. If he is still anxious to try its efficacy, let him make make use of your offer, and test some of those who have passed the rubicon under your care.

I observe in your Treatise, and more lately in the Medical and Physical Journal, you have fallen into an error, respecting the greater rapidity of the disease in the Negro, from some imperfect experiments made in Paris. I find Dr. Marshall also admits the same, from a solitary example noticed by him, (Medical and Physical Journal, for January, page 40). The experience I have had in this disease, in the Negro, does not authorise this conclusion. I have not perceived any difference in the progress of the pock, nor in the facility of its communication, between Whites, Blacks, or Mulattoes. In p. 149 of my Treatise, you will find one solitary instance of its rapid march in a black child; in whom the dark coloured scab was completely formed on the eighth day; and on the eleventh, I tested him with variolous matter. On the fifteenth day the scab came off. The like uniformity has, I believe, been generally observed by our practitioners in Philadelphia. As for the infection being procured so early as the third day, by Dr. Marshall, it is a mere accidental variety of the disease, and not so remarkable as the protracted period you mention of 45 days, in a late Number of the Med. Journal. Case 43 of my Treatise, gives the account of matter obtained from a pock in a white child, in ninety-three hours after vaccination. The usual period of the successful appearance of vaccination is three days, before which time I presume we might always destroy the disease by excision or caustic. I presume this would also be the case in your long-protracted one above adverted to; as I suppose, during this period, the infection must be local, and lie dormant. Now, three days compared with forty-five, is not greater in disproportion, than one month or six weeks, (the usual period of the attack of hydrophobia after a bite) and a year or eighteen months or more, which has been recorded. An important deduction I would wish to draw from this, is, that any time between the reception of the bite, and before the actual occurrence of the symptoms, *excision* should be uniformly practiced; as in all probability the virus must here also be local, and lie dormant. The sooner however this is done, I conceive, the better. The operation of morbid poisons, I think, may be much unfolded, by due attention to this extraordinary blessing.

I see, Mr. Goldson has availed himself of the supposed superiority of size of the natural vaccine-pock over that induced induced by vaccination. This also, I apprehend, is a mere accidental circumstance; I doubt much, whether Mr. Goldson has ever seen a pock on the cow, or in a human subject, of the magnitude of that which I sent you an account of some time ago. The infection, which produced this pock of full an inch and a half in diameter, in a mulatto woman, excited only a common sized pock in another case. It is these variations from the natural appearance of the disease which we must study, in order to perfect our knowledge of this wonderful prophylactic.

I have thus, my dear Sir, encroached upon your valuable time, by a much longer letter than I intended, when I sat down to write. The importance of the subject has however prolonged my communication; and, knowing the interest you feel in whatever concerns it, I trust you will pardon me. If you think it may be useful to communicate these remarks to the Medical and Physical Journal, you have my free consent.

Be assured I am, very truly,

Your obliged humble servant,

JOHN REDMAN COXE.

P.S. I must request your acceptance of the first Number of the Philadelphia Medical Museum; a work I have lately undertaken to conduct here. If you would occasionally enrich its pages by some of the stores of your information, I shall be highly flattered. You will, in this Number, see some remarks on the subject of vaccination; and you will also notice the recommendation of the burnt sponge, from your authority. As we have, in various parts of America, districts afflicted with bronchocele, I hope we shall be enabled to give it a complete trial. You will observe the correction of a mistake of the quantity of the sponge in each troche, which has, somehow, been admitted in your communication to the Medical Journal.

